# Development of an Immune-Related Prognostic Signature in Breast Cancer

**DOI:** 10.3389/fgene.2019.01390

**Published:** 2020-01-28

**Authors:** Peiling Xie, Yuying Ma, Shibo Yu, Rui An, Jianjun He, Huimin Zhang

**Affiliations:** ^1^Department of Breast Surgery, the First Affiliated Hospital of Xi’an Jiaotong University, Xi’an, China; ^2^Department of Structural Heart Disease, the First Affiliated Hospital of Xi’an Jiaotong University, Xi’an, China; ^3^Department of Anesthesiology, the First Affiliated Hospital of Xi’an Jiaotong University, Xi’an, China

**Keywords:** breast cancer, immune system, model statistical, prognosis, survival

## Abstract

**Background:**

Although increased early detection, diagnosis and treatment have improved the outcome of breast cancer patients, prognosis estimation still poses challenges due to the disease heterogeneity. Accumulating data indicated an evident correlation between tumor immune microenvironment and clinical outcomes.

**Objective:**

To construct an immune-related signature that can estimate disease prognosis and patient survival in breast cancer.

**Methods:**

Gene expression profiles and clinical data of breast cancer patients were collected from The Cancer Genome Atlas (TCGA) and Gene Expression Omnibus (GEO) databases, which were further divided into a training set (n = 499), a testing set (n = 234) and a Meta-validation set (n = 519). In the training set, immune-related genes were recognized using combination of gene expression data and ESTIMATE algorithm-derived immune scores. An immune-related prognostic signature was generated with LASSO Cox regression analysis. The prognostic value of the signature was validated in the testing set and the Meta-validation set.

**Results:**

A total of 991 immune-related genes were identified. Twelve genes with non-zero coefficients in LASSO analysis were used to construct an immune-related prognostic signature. The 12-gene signature significantly stratified patients into high and low immune risk groups in terms of overall survival independent of clinical and pathologic factors. The signature also significantly stratified overall survival in clinical defined groups, including stage I/II disease. Several biological processes, such as immune response, were enriched among genes in the immune-related signature. The percentage of M_2_ macrophage infiltration was significantly different between low and high immune risk groups. Time-dependent ROC curves indicated good performance of our signature in predicting the 1-, 3- and 5-year overall survival for patients from the full TCGA cohort. Furthermore, the composite signature derived by integrating immune-related signature with clinical factors, provided a more accurate estimation of survival relative to molecular signature alone.

**Conclusion:**

We developed a 12-gene prognostic signature, providing novel insights into the identification of breast cancer with a high risk of death and assessment of the possibility of immunotherapy incorporation in personalized breast cancer management.

## Introduction

Breast cancer is the most commonly diagnosed cancer and the leading cause of death from cancer in female worldwide, with the expectation that 2,088,849 new cases will be diagnosed with 626,679 related death in 2018 (Bray et al., 2018). Although increased early detection, diagnosis and treatment have brought a sustained decrease in mortality rate over the past decades, almost all patients who progress to metastatic diseases will have poor outcomes even with multimodality therapy (Emens, 2018; Weigel and Dowsett, 2010). These sobering data highlight the urgent need for innovative approaches to identify patients with high risk disease, and continuously improving the management of breast cancer. At the era of increasing interest in personalized medicine, the role of gene-expression profiling in providing guidance to individual treatment optimization is of considerable importance.

Breast cancer is not a single disease, but comprises heterogeneous and diverse groups, with patients in the similar stage disease varying in clinicopathological features, response to systemic therapies and clinical outcomes (Blows et al., 2010). Over the past decades, the advancement and widespread application of “omics” has provided important insights into molecular complexity of breast cancer, prompting researchers to systematically explore methods to identify patients with high risk disease better (Xiao et al., 2019). A number of studies suggested that multigene signatures might be more accurate for risk stratification than the conventional approaches in breast cancer (Buyse et al., 2006; Reis-Filho and Pusztai, 2011; Toole et al., 2014). Oncotype DX is a 21-gene signature providing a stratification of distant relapse risk to weigh the benefits of chemotherapy and the risks of side effects (Toole et al., 2014). Another model, MammaPrint, a 70-gene signature, is also a prognostic model to stratify patients with breast cancer into high or low risk group for relapse (Buyse et al., 2006). These multigene assays, to some extent, have already been incorporated into clinical practice, but still awaiting the results of large, randomized trials for the highest level of evidence of utility (Kwa et al., 2017).

Increasing evidence support a vital role of the immune system in breast cancer initiation and development (Emens, 2012; Gonzalez et al., 2018). The evolving interactions between breast tumors and host immunity establish a formidable network of immune tolerance within tumor microenvironment (TME), which allow overt immune escape and tumor progression to occur (Emens, 2018; Gonzalez et al., 2018). Immune checkpoint molecules enhancing suppressive activity are upregulated on tumor cells while immune-suppressive signaling pathways are activated in numerous immune cell types in this process (Emens, 2018). Recent immunotherapies targeting specific immune checkpoint PD-1/PD-L1 have had remarkable success in anti-tumor therapy, inducing durable clinical responses that translate into a survival benefit in some patients with metastatic triple negative breast cancer (TNBC) (Emens, 2018). Additionally, studies of the breast tumor microenvironment suggest that the presence of immune infiltration is a favorable prognostic marker particularly among TNBC, HER2+ and highly proliferative ER positive tumors (Bianchini et al., 2010; Mahmoud et al., 2011). However, the molecular characteristics describing immune interaction remain unknown and need to be comprehensively investigated due to their prognostic potential in breast cancer.

In this study, by using combination of gene expression data and ESTIMATE algorithm-derived immune scores, we aimed to recognize a group of immune-related genes and construct a reliable multi-gene-based prognostic model for breast cancer patients.

## Materials and Methods

### Database

The UCSC Xena web-based data mining platform (https://xena.ucsc.edu/) was used to download RNA-seq data from The Cancer Genome Atlas (TCGA). Gene expression profiles of 733 breast cancer samples were obtained for TCGA dataset, along with clinical data such as age, molecular subtype, survival and outcome. Immune scores were calculated to the dataset above by applying the ESTIMATE-a method that predicts the extent of immune cell infiltration in tumor samples by analyzing a specific gene signature related to immune cells (Yoshihara et al., 2013). Among the TCGA cohort, 733 patients with relevant information were randomized and divided into a training set (n = 499) and a testing set (n = 234). To validate the prognostic value of the gene signature discovered from the training set, three gene expression array (GSE20685, GSE20711 and GSE42568) containing 519 breast cancer patients and their corresponding clinical information were retrieved from the Gene Expression Omnibus (GEO) (https://www.ncbi.nlm.nih.gov/geo/).

### Identification of IRGs Between Low and High Immune Score Groups

The training set containing 499 patients from TCGA cohort was divided into top and bottom halves (high vs. low immune score group) based on their immune scores. LIMMA analysis was carried out to identify the immune-related genes (IRGs) by comparing the normalized expression data between high and low immune score groups. The genes that met the cutoff criteria of a |logFC| > 1 and an adjusted *P* value < 0.01 were considered as IRGs. Hierarchical clustering analysis was performed to show expression patterns of the IRGs between high and low immune score groups.

### Construction of a Prognostic Signature Based on IRGs

The univariate Cox regression analysis was applied to investigate the association between each IRG and overall survival (OS) of patients in the training set. Those genes with P-value < 0.05 were considered as candidate survival-associated IRGs to build the immune-related risk model. To minimize the risk of overfitting, LASSO regression was employed by 10-fold cross-validation to obtain the most strongly survival-associated IRGs in the training set, further fitting in Cox multivariate regression model to generate the regression coefficient for each gene. The immune-related gene prognostic index (IRGPI) is the sum of products of the expression level of each gene and its corresponding regression coefficient, $\text{IRGPI }=\sum_{i=1}^{n}\beta_{i}Exp_{i}$. Using the aforementioned formula, we calculated the IRGPI for each patient in the training set, and a threshold for high and low immune risk groups was chosen manually at the 50th percentile.

### Validation of the Immune-Related Gene Signature

The prognostic value of IRGPI was performed between high and low immune risk groups in the training set, the testing set and the Meta-validation set by Kaplan−Meier survival curve. We then combined IRGPI with available clinical and pathologic features in multivariate analysis to further investigate whether IRGPI was an independent prognostic factor. Age and stage were regarded as continuous variables. Stage was coded as Stage I (1), Stage II A(2), the range between II A and II B(2.5), Stage II B(3), Stage III A(4), Stage III B(5), the range between III A and III C(5), Stage III C(6), Stage IV (7). The prognostic accuracy of IRGPI was measured using concordance index (C-index), which ranges from 0 to 1.0, with 0.5 indicting random chance and 1.0 indicating perfect discrimination. Stratified analysis was performed to further explore the influence of other factors on IRGPI. In addition, we compared the predictive efficiency of IRGPI with the commercially available molecular model Oncotype Dx and other two immune signatures in the literatures by receiver operating characteristic curve (ROC) analysis at 1-year, 3-year, and 5-year OS.

### Profiling of Infiltrating Immune Cells

CIBERSORT, a versatile computational approach for quantifying cell fractions from gene expression data of bulk tissue, was applied to analyze the abundance of diverse immune cell types in different risk groups (Newman et al., 2015). Customized function in CIBERSORT web portal (http://cibersort.stanford.edu/) enabled us to uploaded normalized gene expression data for analysis, with default matrix at 1000 permutations. As a result, the proportion of 22 types of infiltrating immune cells, including T cells, B cells, macrophages, neutrophils, dendritic cells, and others, were calculated for each sample. TIMER, a similar web-based data mining platform (https://cistrome.shinyapps.io/timer/), was used to reversely verify the results from CIBERSORT.

### Gene Ontology (GO) Analysis

The g:Profiler web-based platform (https://biit.cs.ut.ee/gprofiler/) was used to gain an insight into functional enrichment of genes involved in the immune-related signature. FDR-adjusted P value < 0.05 was set as the cutoff to screen significant gene sets.

### Construction and Validation of a Composite Immune-Clinical Prognostic Index

To refine the risk model, age, stage and IRGPI were integrated into immune-clinical prognostic index (ICPI) by using Cox proportional hazards regression in the training set. The procedure for risk stratification by ICPI was repeated as IRGPI. Further, we compared the predictive efficiency of ICPI with that of IRGPI in terms of C-index, which was depicted by restricted mean survival (RMS) curve. Similarly, the cutoff value for ICPI was set manually at the 50th percentile to keep consistent with IRGPI.

### Statistical Analysis

All statistical tests were performed using R (version 3.6.0; https://www.r-project.org/) and SPSS (version 19.0; SPSS Company, Chicago, IL). Continuous variables were analyzed with the t-test or the Wilcoxon rank sum test while categorical variables were compared with the Pearson Chi-square test. The immune scores were calculated by ESTIMATE package. Univariate and multivariate Cox proportional hazards regression were performed by survival package. Kaplan−Meier curve and RMS curve were also performed by survival package. LASSO Cox proportional hazards regression analysis was performed by penalized package. Time-dependent ROC curve was done with TimeROC package. The C-index was calculated and compared with survcomp package. The PAM50 subtype and Oncotype Dx recurrence scores were estimated with genefu package. For all tests, a two-sided P-value < 0.05 was considered statistically significant.

## Results

### Immune Scores Are Significantly Associated With Breast Cancer PAM50 Subtypes

**Figure 1:**
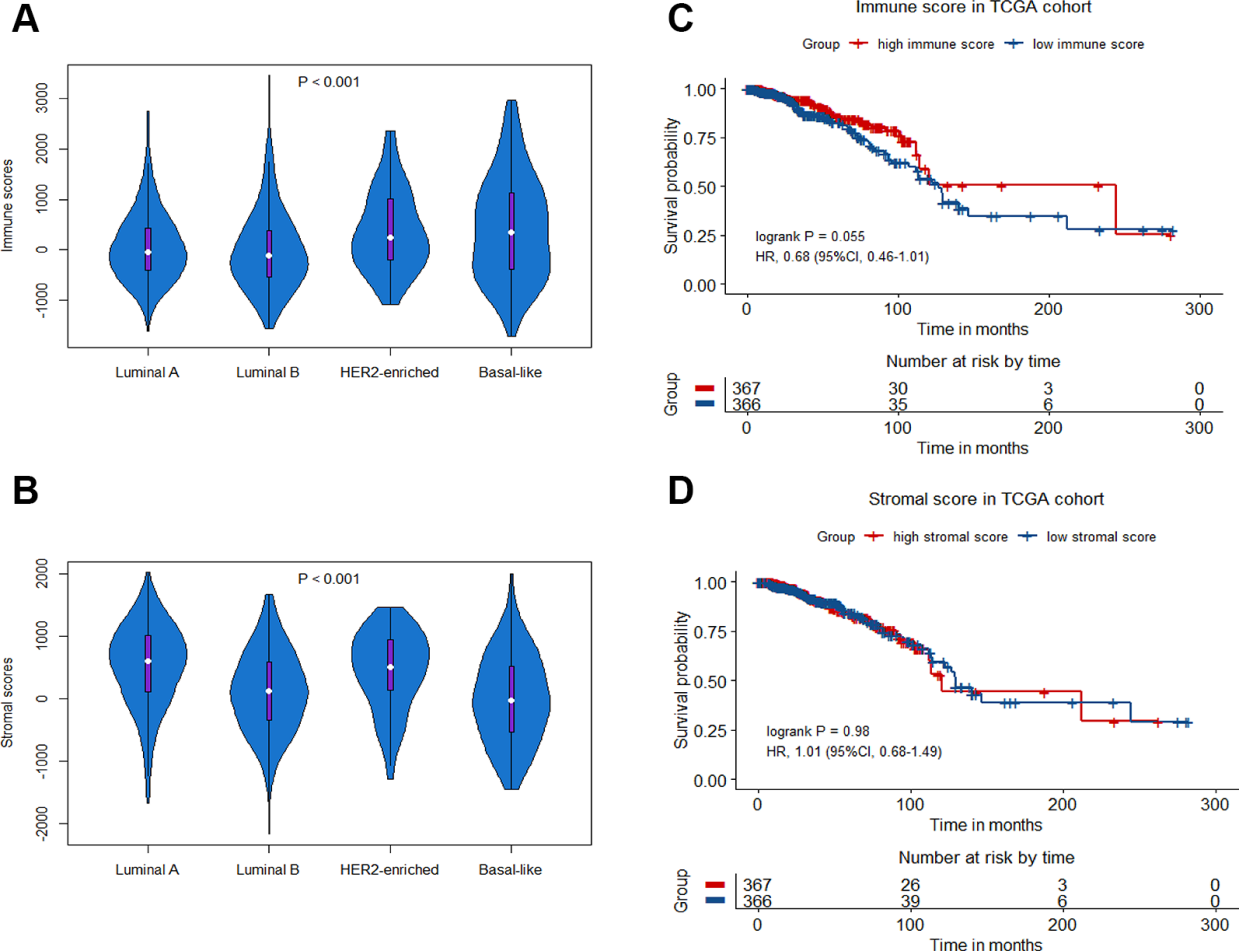
Immune scores and stromal scores in breast cancer. Violin-plot showing the distribution of immune scores **(A)** and stromal scores **(B)** among different breast cancer molecular subtypes. Kaplan−Meier curves of overall survival (OS) among high or low risk groups based on immune scores **(C)** and stromal scores **(D)** in the TCGA cohort.

A total of 1252 patients with complete gene expression data and relevant information were included in the analysis. TCGA cohort was randomly divided into the training set and the testing set while GSE20685, GSE20711 and GSE42568 as the Meta-validation set (**Table 1**). To characterize the role of immune scores and stromal scores in breast cancer, we calculated the immune score and the stromal score by ESTIMATE algorithm for each patient from TCGA cohort. The immune scores ranged from -1724.49 to 3459.35, and stromal scores were distributed between −2164.14 and 2026.84. The average immune score of basal-like subtype cases ranked the highest of all four subtypes (except normal-like in PAM50 subtype), followed by HER2-enriched subtype, and luminal subtype (*P* < 0.001) (**Figure 1A**). The rank order of stromal scores from highest to lowest is luminal A > HER2-enriched > luminal B > basal-like (*P* < 0.001) (**Figure 1B**). To explore the potential correlation between OS and immune/stromal scores, patients were divided into low and high score groups based on their scores, with median immune/stromal score as the cutoff value. Kaplan−Meier survival curve indicated that patients in high immune score group were likely to achieve more favorable outcome than those in low immune score group (HR = 0.68, 95%CI = 0.46-1.01, P = 0.055) (**Figure 1C**), while cases in high and low stromal score group showed no significant difference in OS (HR = 1.01, 95%CI = 0.68-1.49, P = 0.98) (**Figure 1D**).

**Table 1:** Clinical and Pathologic Feature of Patients in the Training Set, Testing Set, and Meta-validation Set.

Characteristic	TCGA cohort			Meta-validation cohort		
	Training set	Testing set	P-value	GSE20685	GSE20711	GSE42568
No. of samples	499	234		327	88	104
Median age in years (range)	58 (48–66)	58 (48–68)	0.74	48 (47–49)	NA	56 (49–68)
AJCC stage						
I (%)	86 (17)	39 (17)	0.85	69 (21)		
IIA (%)	178 (36)	79 (34)	0.61	147 (45)^†^		
IIB (%)	115 (23)	49 (21)	0.52			
IIIA (%)	77 (15)	40 (17)	0.57	103 (31)^‡^	NA	NA
IIIB (%)	8 (2)	11 (5)	0.01			
IIIC (%)	23 (5)	14 (6)	0.43			
IV (%)	12 (2)	2 (1)	0.25	8 (2)		
PAM50 subtype^§^						
Luminal A (%)	223 (45)	102 (44)	0.78	121 (37)	23 (26)	31 (30)
Luminal B (%)	121 (24)	59 (25)	0.77	80 (24)	22 (25)	42 (40)
HER2-enriched (%)	53 (11)	25 (11)	0.98	68 (21)	21 (24)	15 (14)
Basal-like (%)	95 (19)	41 (18)	0.62	42 (13)	22 (25)	23 (22)
Median follow-up (months)	38	24		113	92	82
No. of death (%)	72 (14)	28 (12)	0.37	83 (25)	25 (28)	35 (34)

^†^Annotated as stage II patients only; ^‡^Annotated as stage III patients only; ^§^Normal-like is not included.

### Construction of an Immune-Related Gene Signature and Its Prognostic Value

**Figure 2:**
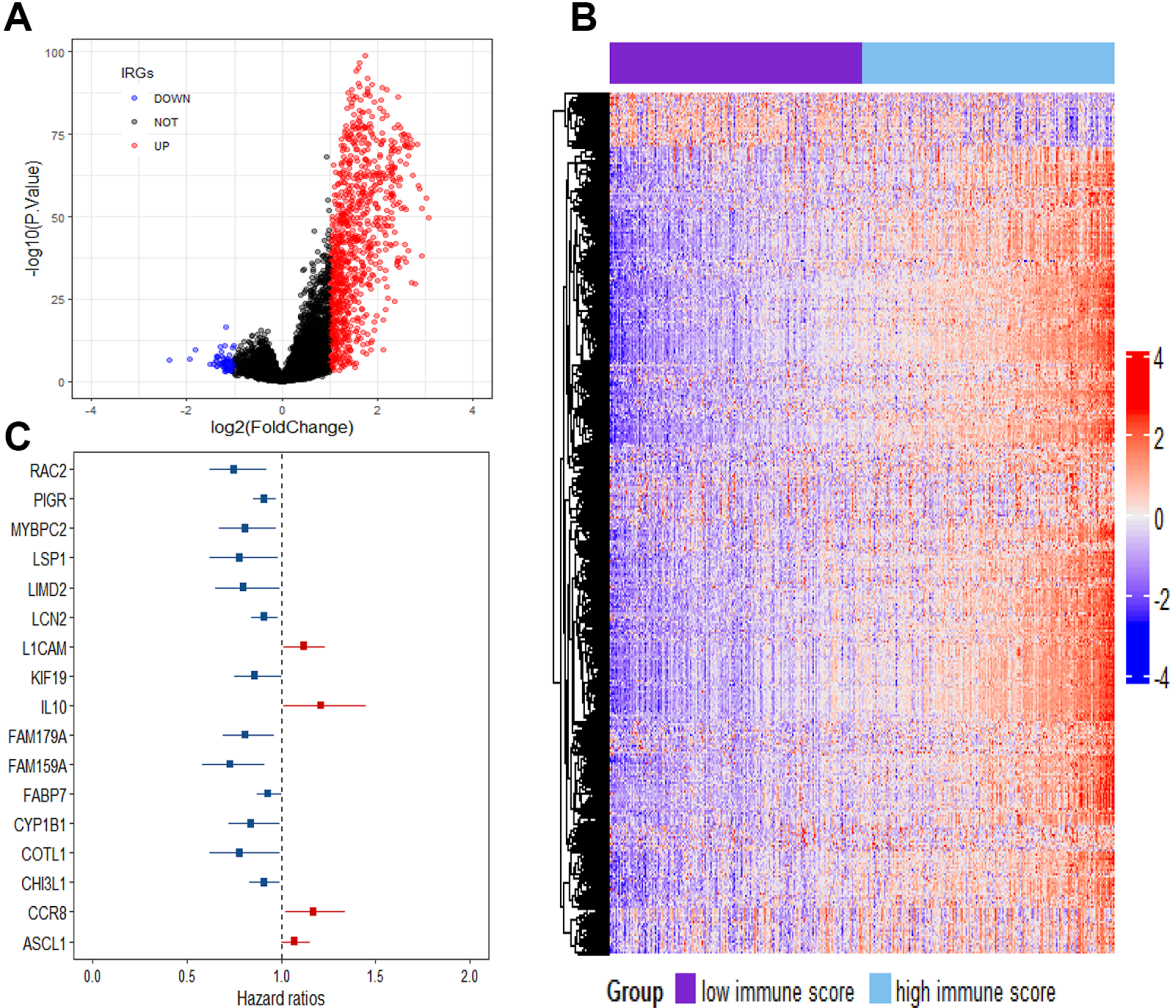
Comparison of gene expression profile with immune scores in breast cancer. **(A)** Volcano plot visualizing the immune-related genes between high and low immune score groups. Red plots represent aberrantly expressed genes with adjusted P-value < 0.01 and log_2_FC > 1. Black plots represent normally expressed genes. Blue plots represent aberrantly expressed genes with adjusted *P* value < 0.01 and log_2_FC < 1. **(B)** Heatmap analysis of the immune-related genes between high and low immune score groups. **(C)** Forest plot of hazard ratios showing the survival-associated immune-related genes.

**Figure 3:**
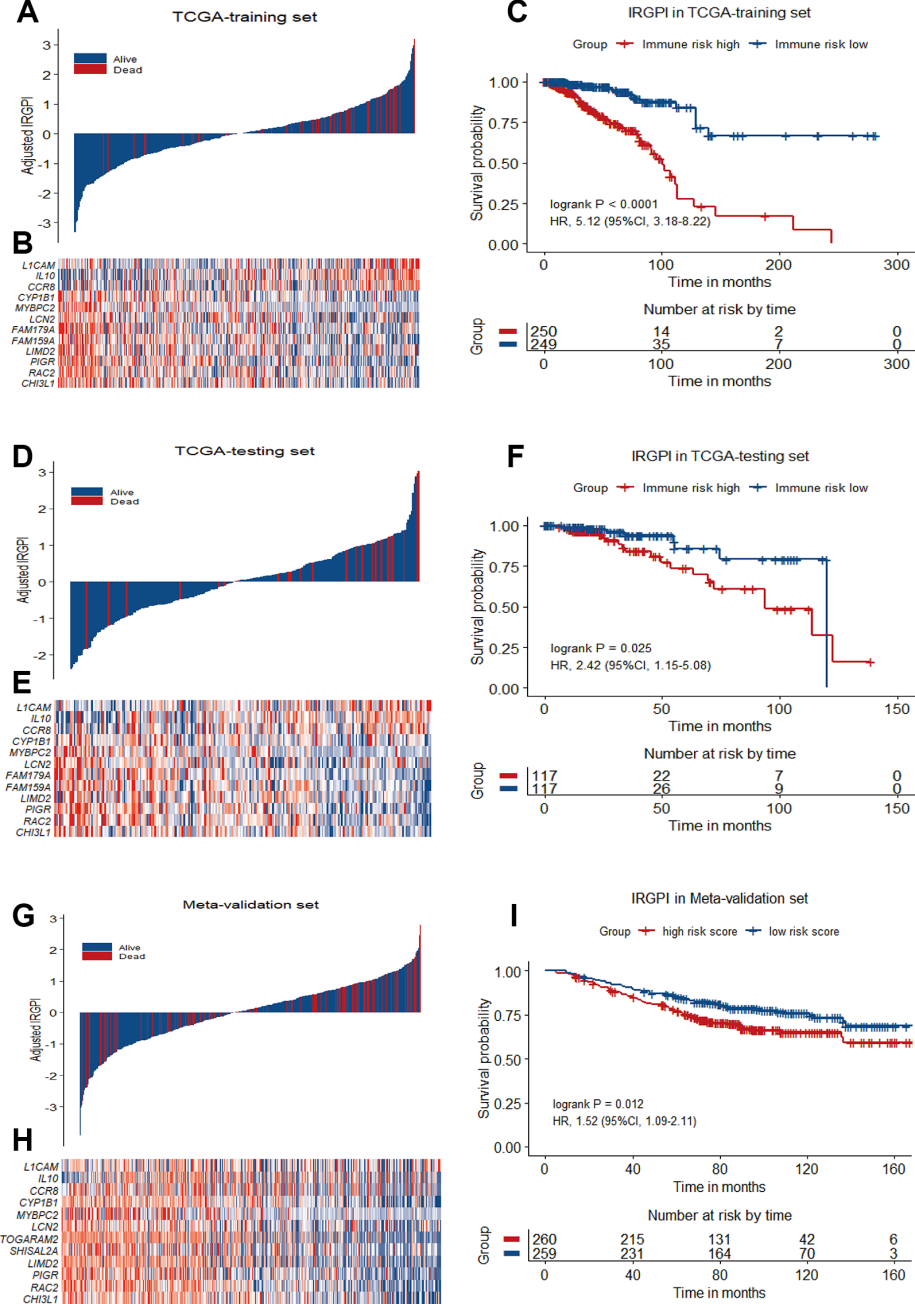
12-gene prognostic signature biomarker characteristics in TCGA training set, TCGA testing set and Meta-validation set. **(A)**, **(D)** and **(G)** Breast cancer were ranked by immune risk scores in the TCGA training set, TCGA testing set and Meta-validation set. **(B)**, **(E)** and **(H)** Heatmap of 12 genes related to IRGPI differentially expressed between high and low immune risk groups in the TCGA training set, TCGA testing set and Meta-validation set, with red indicating higher expression and blue indicating lower expression. Patients were stratified by immune-related gene prognostic index (IRGPI) (low immune risk vs. high immune risk). **(C)**, **(F)**, and **(I)** Kaplan−Meier curves of overall survival (OS) among different IRGPI risk groups in TCGA training set, TCGA testing set and meta-validation set. Hazard ratios (HRs) and 95% CIs are for high immune risk group vs. low immune risk group. P values comparing risk groups were calculated with the log-rank test.

To reveal the correlation of gene expression profiles with immune scores, we compared Illumina RNA-seq data of 499 breast cancer cases in the TCGA training set. A total of 991 IRGs with 927 genes upregulated and 64 genes downregulated were identified in high immune score group when compared with low immune score group (**Figure 2A** and **Table S1**). Heatmap analysis showed that these genes presented differential gene expression profiles between low and high immune score groups (**Figure 2B**). The association of 991 IRGs with OS was assessed using univariate Cox analysis in the training set, and 17 prognostic IRGs were recognized (**Figure 2C**). Then LASSO Cox proportional hazards regression analysis was performed based on the 17 initial candidate prognostic IRGs, and 12 genes were found to be the final prognostic IRGs which were used in IRGPI construction (**Table 2**). The IRGPI was calculated as the following formula: IRGPI = 0.177*LCAM − 0.152*CYP1B1 − 0.111*MYBPC2 − 0.028*LCN2 − 0.256*FAM179A − 0.140*FAM159A − 0.107*LIMD2 − 0.051*PIGR − 0.180*RAC2 + 0.483*IL10 − 0.075*CHI3L1 + 0.149*CCR8. High immune risk group defined by the 12-gene signature-based IRGPI, had significant worse OS (HR = 5.12, 95%CI = 3.18-8.22, P < 0.0001) in the TCGA training set independent of age, stage and molecular subtype (**Figures 3A–C** and **Table 3**).

**Table 2:** Immune-related Gene Signature in the Prognostic Model.

Gene symbol	Full name	Coefficient
L1CAM	L1 cell adhesion molecule	0.177
CYP1B1	Cytochrome P450 family 1 subfamily B member 1	−0.152
MYBPC2	Myosin binding protein C, fast type	−0.111
LCN2	Lipocalin 2	−0.028
FAM179A^†^	Family with sequence similarity 179 member A	−0.256
FAM159A^‡^	Family with sequence similarity 159 member A	−0.140
LIMD2	LIM domain containing 2	−0.107
PIGR	Polymeric immunoglobulin receptor	−0.051
RAC2	Rac family small GTPase 2	−0.081
IL10	Interleukin 10	0.483
CHI3L1	Chitinase 3 like 1	−0.075
CCR8	C-C motif chemokine receptor 8	0.149

^†^Also known as TOGARAM2; ^‡^Also known as SHISAL2A.

**Table 3:** Univariate and Multivariate Analysis of Prognostic Factors in the Training set, Testing set and Meta-validation Set.

Data sets	Variables	Univariate		Multivariate	
		HR (95%CI)	*P* value	HR (95%CI)	*P* value
TCGA-training	Age	1.04 (1.02–1.06)	1.76e-04	1.04 (1.02–1.06)	2.98e-04
	Stage	1.33 (1.17–1.52)	1.69e-05	1.25 (1.10–1.43)	7.68e-04
	PAM50 subtype				
	LumA/LumB	Reference		Reference	
	HER2-enriched	1.90 (0.99-3.70)	0.055	–	–
	Basal-like	0.83 (0.45–1.55)	0.56	–	–
	IRGPI	2.72 (2.09–3.54)	7.99e-14	2.51 (1.92–3.27)	**1.08e-11**
TCGA-testing	Age	1.03 (1.00–1.06)	0.028	1.03 (1.01–1.06)	0.012
	Stage	1.38 (1.07–1.78)	0.012	1.37 (1.05–1.78)	0.019
	PAM50 subtype				
	LumA/LumB	Reference		Reference	
	HER2-enriched	0.97 (0.28–3.29)	0.96	–	–
	Basal-like	0.72 (0.24–2.12)	0.55	–	–
	IRGPI	2.34 (1.48–3.70)	2.6e-04	2.16 (1.34–3.49)	**0.0015**
Meta-validation	Age	–	–	–	–
	Stage	–	–	–	–
	PAM50 subtype				
	LumA/LumB	Reference		Reference	
	HER2-enriched	2.33 (1.59–3.42)	1.41e-05	2.48 (1.69–3.65)	3.53e-06
	Basal-like	1.45 (0.91–2.33)	0.12	–	–
	IRGPI	1.14 (1.04–1.76)	0.001	1.69 (1.2–2.39)	**0.0028**

Bolded values represent significant shifts in plausibility (P value < 0.05).

### Validation of the IRGPI as an Independent Prognostic Factor

To validate the prognostic value of IRGPI derived from the training set, we applied the same formula to the internal testing set (TCGA-testing) and the Meta-validation set (GSE20685, GSE20711 and GSE42568). Using the same IRGPI threshold chosen in TCGA training set, the 12-gene prognostic signature significantly stratified the TCGA testing set for OS (HR = 2.42, 95%CI = 1.15-5.08, P = 0.025) independent of age, stage and molecular subtype (**Figures 3D–F** and **Table 3**). In the Meta-validation set, again using the same threshold chosen in TCGA training set, the 12-gene prognostic signature was also capable to stratify patients for OS significantly (HR = 1.52, 95%CI = 1.09-2.11, P = 0.012) independent of molecular subtype (**Figures 3G–I** and **Table 3**).

### Stratification Analysis

**Figure 4:**
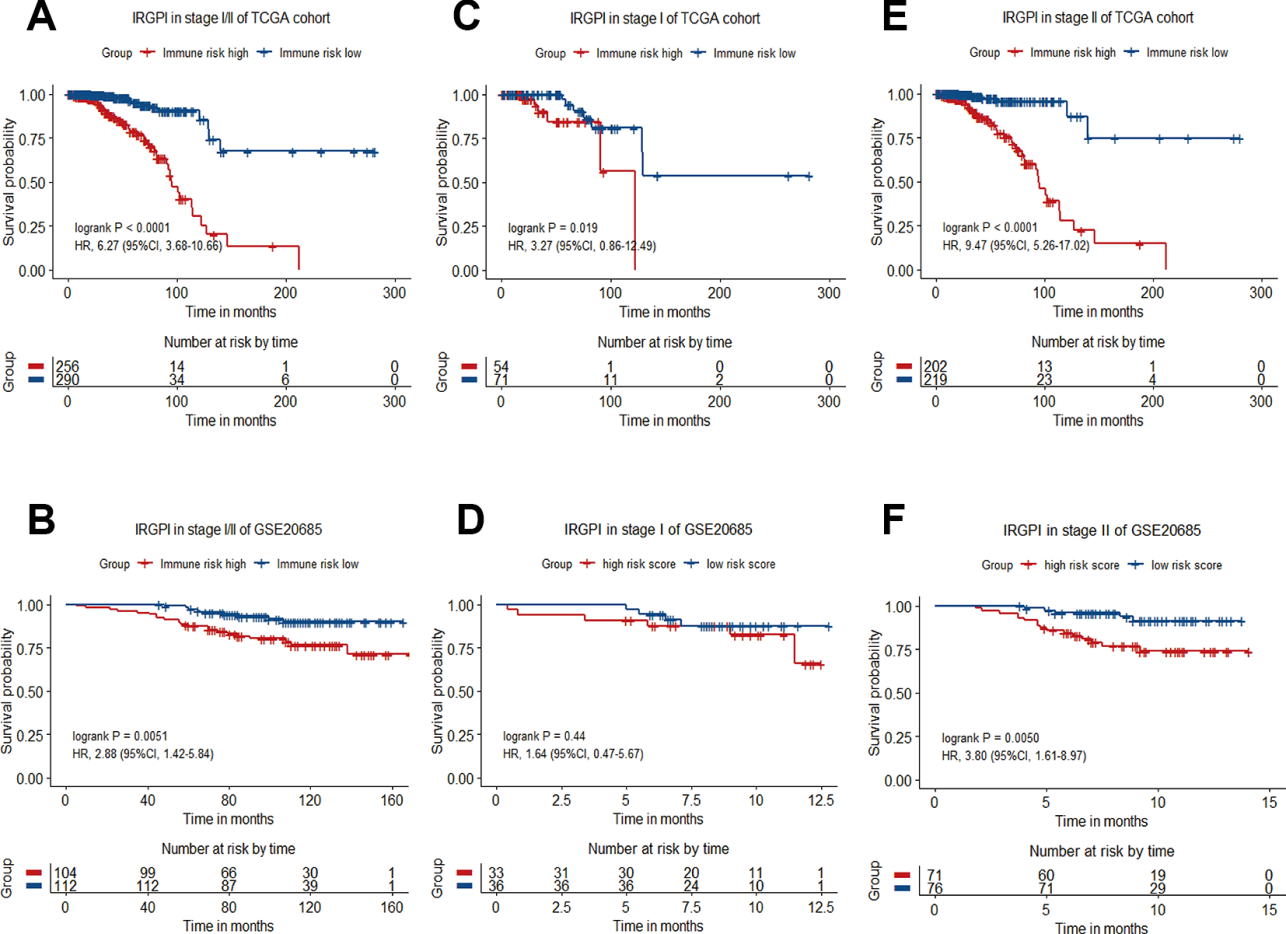
Kaplan−Meier curve of overall survival (OS) for breast cancer patients with different IRGPI risks. **(A)** Survival curve of stage I/II patients of TCGA cohort. **(B)** Survival curve of stage I/II patients in GSE20685 dataset. **(C)** Survival curve of stage I patients of TCGA cohort. **(D)** Survival curve of stage I patients of GSE20685 dataset. **(E)** Survival curve of stage II patients of TCGA cohort. **(F)** Survival curve of stage II patients of GSE20685 dataset.

Breast cancer is a heterogeneous disease that can be classified by a variety of clinical and pathological features, which are routinely used as prognostic factors. The classification of breast cancers into different subgroups may help to predict outcome and choose proper treatment. For stratification analysis, we evaluate the prognostic value of the 12-gene signature in stage-specific groups from the TCGA cohort and the validation dataset. The IRGPI stratified stage I/II breast cancers of TCGA cohort (HR = 6.27, 95%CI = 3.68-10.66, P < 0.0001) and GSE20685 dataset (HR = 2.88, 95%CI = 1.42-5.84, P = 0.0051) into different prognostic subgroups in term of OS significantly (**Figures 4A**, **B**). When further restricted to patients with stage II disease, the IRGPI remained significantly prognostic for TCGA cohort (HR = 9.47, 95%CI = 5.26-17.02, P < 0.0001) and the GSE20685 dataset (HR = 3.80, 95%CI = 1.61-8.97, P = 0.0050) (**Figures 4C–F**).

### Comparison With Other Gene Signatures

**Figure 5:**
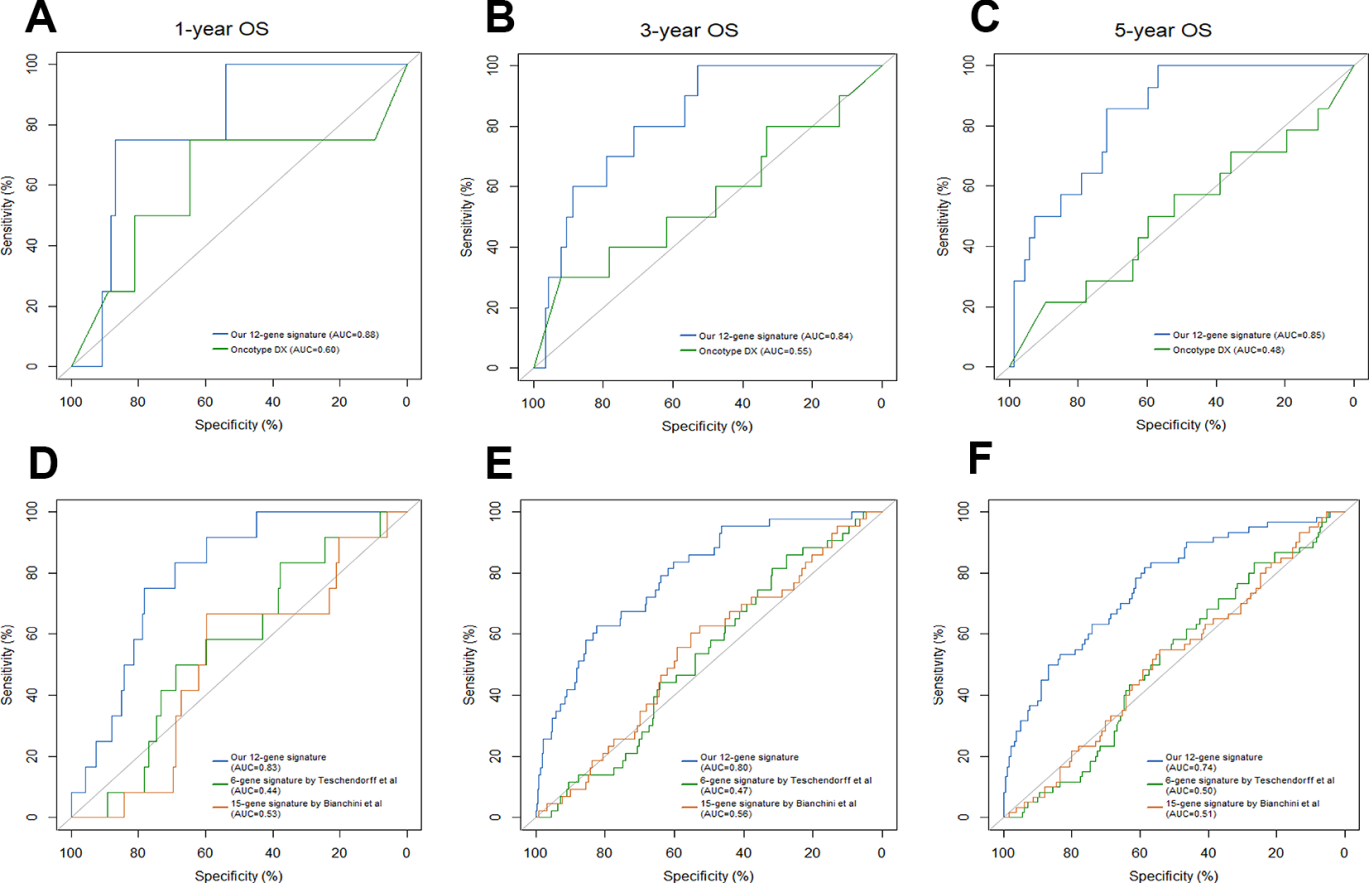
Comparison of our 12-gene signature and other models. Time-dependent ROC analysis was performed to compare our 12-gene signature and Oncotype Dx in predicting 1-year **(A)**, 3-year **(B)** and 5-year **(C)** overall survival (OS). Time-dependent ROC analysis was performed to compare our 12-gene signature and other two immune-related signatures in predicting 1-year **(D)**, 3-year **(E)** and 5-year **(F)** overall survival (OS).

Breast cancer prognostic signatures mainly focus on the patients of early stage as a subset which adjuvant chemotherapy might be tailored based on the risk of recurrence, since most patients in this subset may be adequately treated with endocrine therapy alone (EBCTCG, 2011; Sparano et al., 2015). Previous studies have shown that the 21-gene signature provides information of the likelihood of recurrence and the potential benefit from chemotherapy in ER-positive and node-negative breast cancer (Melisko, 2005; Paik et al., 2006). In reference to the 21-gene signature, NCCN guidelines report that 21-gene recurrence score (RS) can be considered in patients with 1-3 involved lymph nodes to guide additional chemotherapy (Goetz et al., 2019). We compared the 12-gene signature-based IRGPI with the 21-gene signature-based RS in patients with ER/PR-positive, HER2-negative, node-negative or 1-3 involved lymph nodes from the full TCGA cohort. The IRGPI achieved a better accuracy in predicting 1-year, 3-year and 5-year OS (AUC = 0.88, 0.84, 0.85, respectively) than RS (AUC = 0.60, 0.55, 0.48, respectively) (**Figures 5A–C**).

Traditionally, prognostic multigene model strictly consisted of proliferation-associated genes; however, these prognostic models were only highly applicable in ER/PR-positive and node-negative breast cancer subtypes. Recent preclinical studies have demonstrated that inflammation and the immune landscape are essential drivers of breast cancer. Therefore, prognostic multigene signatures based on immune-related genes have emerged, including Teschendorff's 6-gene model, Bianchini's 15-gene model and others (Bianchini et al., 2010; Teschendorff et al., 2010). When compared with Teschendorff's (AUC = 0.44, 0.47, 0.50, respectively) and Bianchini's (AUC = 0.53, 0.56, 0.51, respectively) immune-related signatures, our twelve-gene signature (AUC = 0.83, 0.80, 0.74, respectively) exhibited a higher accuracy in predicting 1-year, 3-year and 5-year OS of breast cancer patients (**Figures 5D–F**).

### IRGPI Is Predictive Factor of pCR to Neoadjuvant Chemotherapy

**Figure 6:**
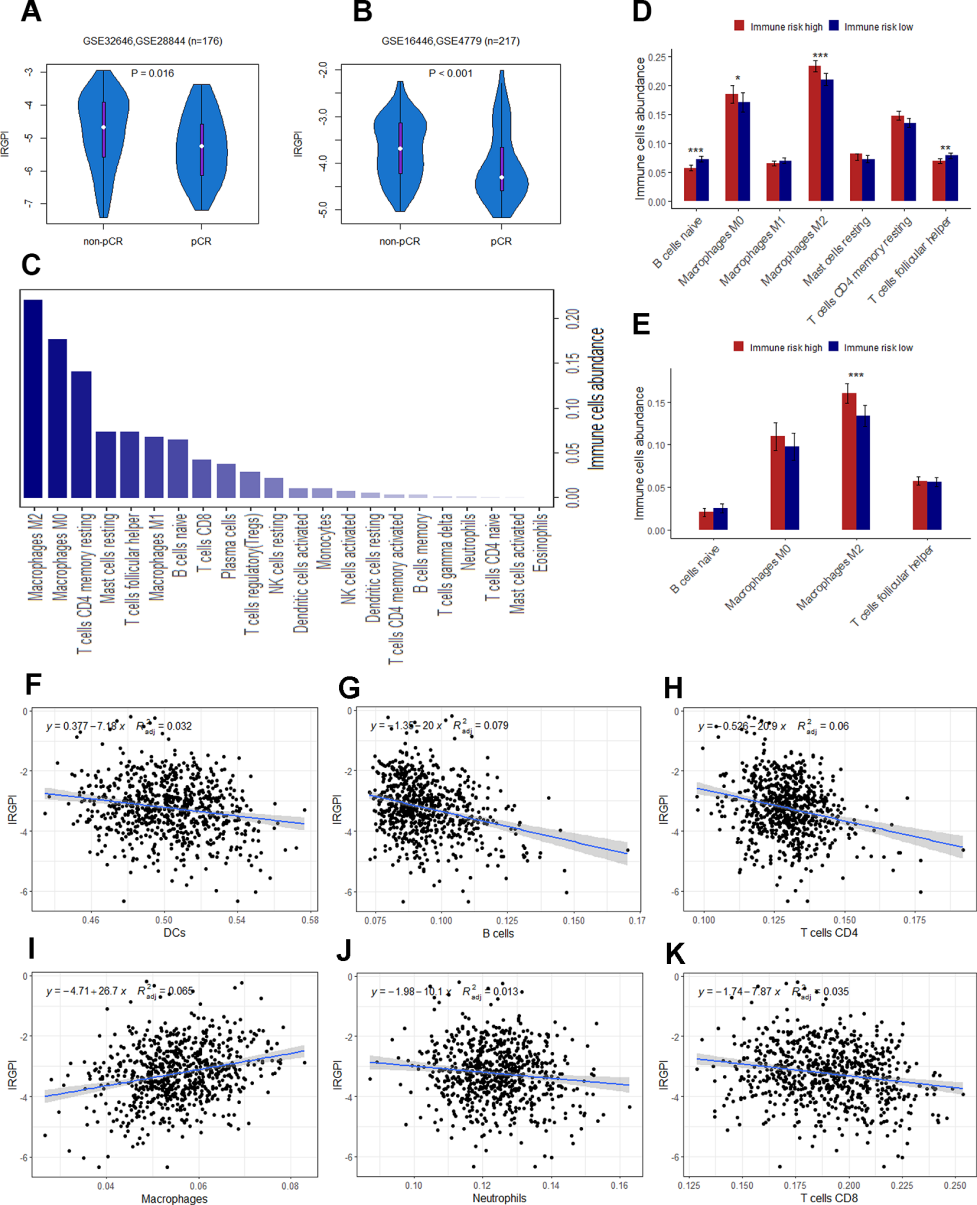
**(A)** The difference of IRGPI between pCR and non-pCR groups in GSE32646 and GSE28844. **(B)** The difference of IRGPI between pCR and non-pCR groups in GSE16446 and GSE4779. **(C)** 22 types of immune cells abundance calculated by CIBERSORT in TCGA cohort. **(D)** Immune cells abundance between low and high immune risk groups for TCGA cohort. **(E)** B cells naïve, Macrophages M_2_, T cells follicular helper and Macrophages M_0_ abundance in GSE20685 dataset. The association between adjusted IRGPI and the abundance of DCs **(F)**, B cells **(G)**, T cells CD4 **(H)**, macrophages **(I)**, neutrophils **(J)**, and T cells CD8 **(K)** calculated by TIMER in TCGA cohort. *P* values are based on Wilcoxon rank sum test. Error bars indicate estimated 95%CI.

The presence of infiltrating immune cells in the breast tumor, and a linear relationship between immune cells infiltrates and clinical outcomes have now been confirmed in large study cohorts (Emens, 2018). A number of studies have also found that the expression of immune-related genes is associated with a better prognosis, and a greater likelihood of pathologic complete response (pCR) to neoadjuvant chemotherapy (Ignatiadis et al., 2012; Asano et al., 2016). Thus, we next seek to assay whether IRGPI could predict pCR to chemotherapy. Patients were divided into pCR and non-pCR subgroups described in the original publications. To balance the sample size of the two groups, we integrated GSE32646 and GSE28844 (GSE16446 and GSE4779) into a new cohort for analysis. In consistent with previous studies, the patients in pCR group had a significantly lower IRGPI than the patients in no-pCR group (**Figures 6A, B**).

### Biological Function Prediction of the Immune-Related Signature

Immune infiltration profiling and GO analysis were carried out to explore the potential biological function of the 12 genes involved in immune-related risk model. For immune infiltrates, such as M_2_ macrophages, M_0_ macrophages, resting memory CD4+ T cells, resting mast cells, follicular T-helper cells, M_1_ macrophages and naïve B cells were enriched in TCGA cohort (**Figure 6C**). We also found that the abundance of naïve B cells, M_2_ macrophages, follicular T-helper cells and M_0_ macrophages were significantly different between low and high immune risk groups in TCGA cohort (**Figure 6D**). Furthermore, the difference of specific immune cells infiltration between two groups was validated in the independent validation set. The mean level of M_2_ macrophages in the high immune risk group was significantly higher than that in low immune risk group in the validation set (**Figure 6E**). In addition, the proportion of immune cell types (B cells, CD4+ T cells, CD8+ T cells, neutrophils, macrophages and dendritic cells) of tumor sample in the TCGA cohort was calculated by TIMER for reverse analysis. As expected, TIMER has drawn the consistent conclusion: patients with higher levels of immune cells infiltration are inclined to lower immune risk (**Figures 6F–K**). The results of GO analysis indicated that 12 genes were mostly enriched in the immune processes such as immune response, leukocyte activation, cell adhesion and so on (**Table 4**).

**Table 4:** Biological processes of genes consisting of IRGPI.

GO term	Description	*P* value
GO:0002274	Myeloid leukocyte activation	9.171 × 10^−3^
GO:0002366	Leukocyte activation involved in immune response	1.326 × 10^−2^
GO:0002263	Cell activation involved in immune response	1.363 × 10^−2^
GO:0070301	Cellular response to hydrogen peroxide	1.858 × 10^−2^
GO:0007155	Cell adhesion	2.187 × 10^−2^
GO:0022610	Biological adhesion	2.250 × 10^−2^
GO:0002443	Leukocyte mediated immunity	3.847 × 10^−2^

### Integrated Risk Score by Combining the IRGPI With Clinical Factors

**Figure 7:**
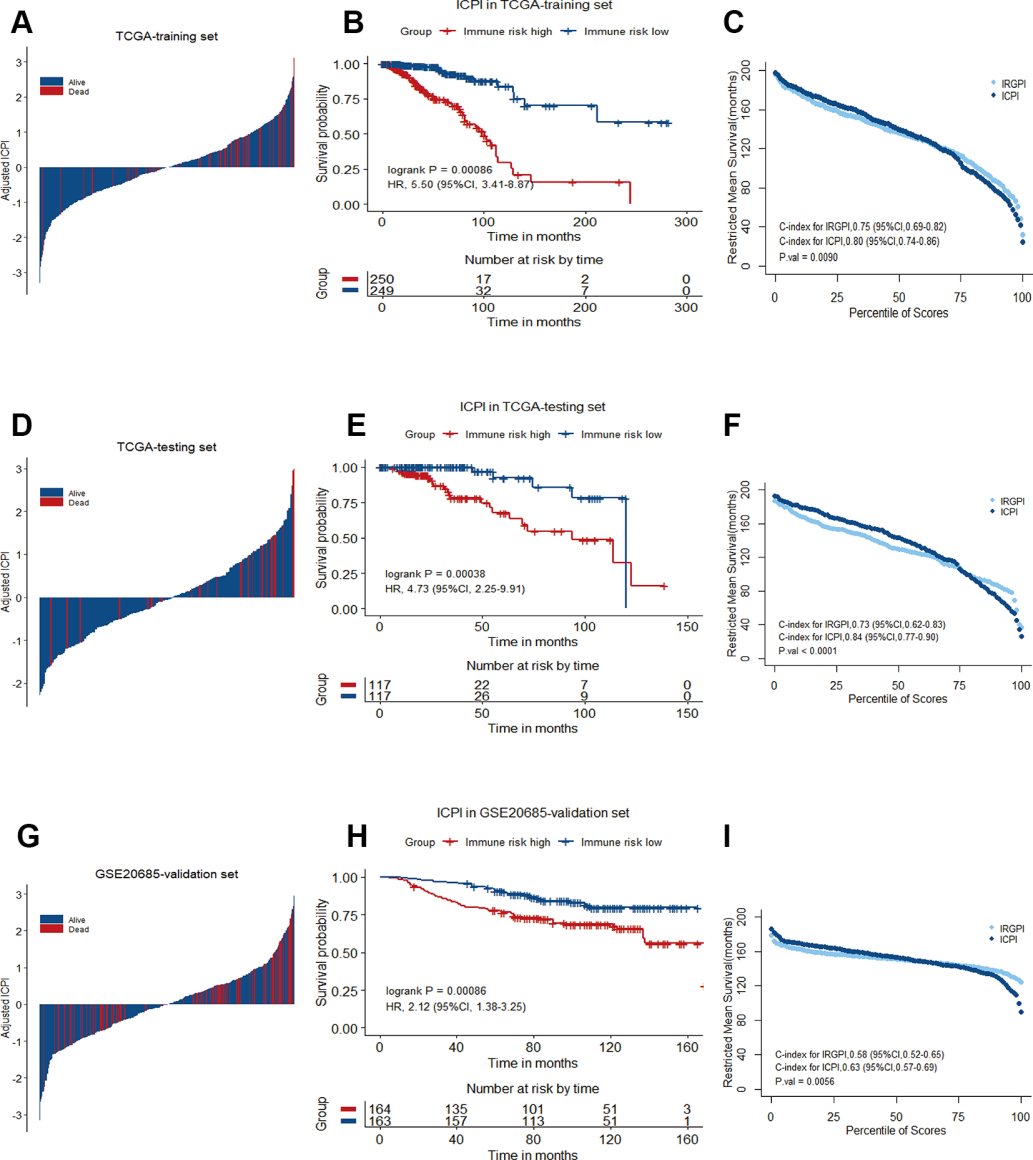
Immune-clinical prognostic signature characteristics in TCGA training set, TCGA testing set and GSE20685 validation set. **(A)**, **(D)**, and **(G)** Breast cancer were ranked by immune-clinical risk scores in the TCGA training set, TCGA testing set and GSE20685 validation set. **(B)**, **(E)**, and **(H)** Kaplan−Meier curves of overall survival (OS) among different ICPI risk groups in TCGA training set, TCGA testing set and GSE20685 validation set. Hazard ratios (HRs) and 95% CIs are for high vs. low immune risk. P values comparing risk groups were calculated with the log-rank test. **(C)**, **(F)**, and **(I)** Restricted mean survival curves for IRGPI and ICPI scores was plotted on TCGA training set, TCGA testing set and GSE20685 validation set.

In multivariate Cox analysis, age, stage and IRGPI remained as independent prognostic factors after adjusted by clinicopathological factors in at least 2 datasets. To further improve accuracy, age, stage and IRGPI were used to fit a Cox proportional hazards regression model in the training set and derive an ICPI as 0.036*age + 0.225*stage + 0.920*IRGPI. By the continuous form of ICPI, the estimation of survival was significantly improved compared with IRGPI (mean C-index, 0.80 vs. 0.75 in the training set, P = 0.0090) (**Figure 7**).

## Discussion

**Figure 8:**
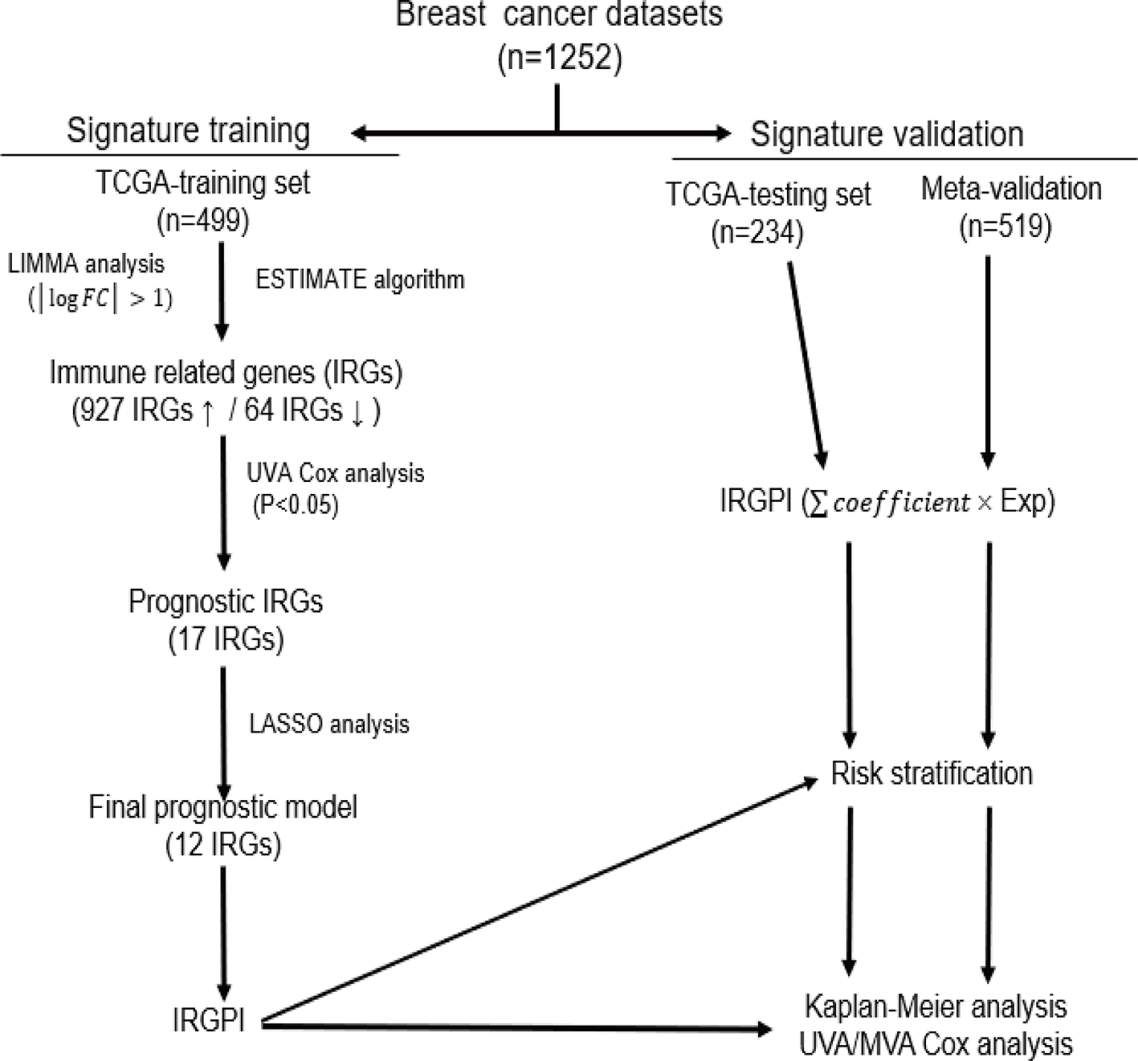
The workflow of construction and validation of our immune-related prognostic signature.

Breast cancer is a heterogeneous group of diverse subtypes, each with its own biologic and clinical characteristics (Blows et al., 2010). Increasing interests aroused on finding reliable prognostic biomarkers to better identify patients with high risk disease, who would benefit from intensive treatment. In an effort to bolster clinical tools for immunobiologic understanding in breast cancer, we developed a prognostic signature associated with tumor immune microenvironment. Based on ESTIMATE algorithm, we calculated immune score for each patient from TCGA breast cancer cohort. The rank order of immune scores across molecular subtypes from highest to lowest is basal-like > HER2-enriched > luminal. This is consistent with previous studies that TNBC and HER2+ breast cancers are more likely to harbor immunogenicity which suggests potential benefits from immunotherapy than luminal breast cancers (Emens, 2018; Savas et al., 2016; Xiao et al., 2019). Genes that were differentially expressed between high and low immune score groups were considered as IRGs for further survival analysis, and 17 IRGs with statistical significant prognostic association were found. Prognostic model training in the TCGA training set selected a 12-gene signature, which was significantly associated with OS and further validated both in the TCGA testing set, and the Meta-validation set (**Figure 8**). Thus, our 12-gene signature provides new perspectives for the identification of breast cancer with a high risk of death and assessment of the possibility of immunotherapy incorporation in personalized breast cancer management.

Our analysis is also likely to provide biological and therapeutic information. IL10 is considered an immunosuppressive cytokine with a crucial role as a feedback regulator of diverse immune response (Saraiva and O'Garra, 2010). The upregulation of IL10 in tissue and serum was observed in triple-negative breast cancer patients and strongly correlated with increased tumor stage and poor outcome. It is the most studied gene of the twelve, being nominated as therapeutic target for pancreatic and breast cancers (Shen et al., 2018). L1CAM, an axonal glycoprotein belonging to immunoglobulin superfamily, is associated with poor prognosis and metastases formation in several cancers, including breast and pancreas. Over-expression of L1CAM not only induced NF-κB activation but also mediated the phosphorylation of FAK and Src, which promoted cancer cell proliferation and tumor growth (Kiefel et al., 2012; Nakaoka et al., 2017). LCN2 is a neutrophil gelatinase-associated lipocalin and plays a role in innate immunity by limiting bacterial growth. It was reported that LCN2 promoted breast cancer progression by inducing epithelial to mesenchymal transition (EMT) through the ERα/Slug axis and might represent a biomarker of breast cancer (Yang et al., 2009). LIMD2 is a mechanistically undefined LIM-only protein that was originally found to be expressed in metastatic lesion. Experimental data showed that LIMD2 linked with integrin-mediated signaling to cell motility and metastatic behaviors in bladder, breast and thyroid tumors (Peng et al., 2014). RAC2 is part of the small Rho GTPase superfamily and is specifically expressed in hematopoietic cells. RAC2 signaling events were proved to be relevant with phospholipase D-induced breast cancer cell invasion and found to regulate actin cytoskeleton during breast cancer metastasis (Henkels et al., 2013; Li et al., 2013). CHI3L1, a highly evolutionary conserved secreted protein, was recently shown to be involved in facilitating tumor progression and metastasis by triggering the MAPK and PI3K signaling pathways in macrophages (Chen et al., 2017; Cohen et al., 2017). CCR8 is a member of chemokine receptor family and plays a pivotal role in recruitment of regulatory T (T_reg_) cells to tumor by the CCL1-CCR8 axis. CCR8 expression was found to be upregulated in T_reg_ cells and correlated with poor survival of patients with breast cancer (Wang et al., 2019). CYP1B1, an extrahepatic enzyme, plays a physiological role in degradation of 17β-estradiol into carcinogenic 4-hydroxy-estradiol. Over-expression of CYP1B1 has been shown to correlate with Wnt5/6-β-catenin signaling, stem cell phenotype and poor clinical prognostic factors in inflammatory breast cancer (Mohamed et al., 2019). PIGR, a Fc receptor family member, is a central component of the mucosal immune system. Previous studies revealed a pro-oncogenic role of PIGR in hepatocellular cancer by activating Src family tyrosine kinase (Yes) and MEK/ERK signaling (Yue et al., 2017), while PIGR in breast cancer was rarely reported. Moreover, we are the first to report the prognostic value of MYBPC2, FAM159A, and FAM179A in breast cancer, which may provide novel directions for further investigation.

The incidence rates of breast cancer have been rising over the last decades with increases in screening and awareness, of which 70% will be early-stage (stage I–II) disease (Kwa et al., 2017). The traditional methods of the risk stratification for breast cancer use standardized clinical and pathological characteristics to guide therapeutic decision. Those classical features indicating patient prognosis include tumor size, lymph node status, grade and subtype, which could be efficiently utilized to certain subpopulations, however, the prognostic factors used have limitations in predicting individual clinical outcome. For example, patients with resembling clinicopathological features may have distinct outcomes. Many patients with early stage breast cancer would therefore be overtreated with chemotherapy on the basis of clinicopathologic features alone, particularly those with ER/PR positive and HER2 negative tumors. Approximately 60% of patients with early stage breast cancer suffer from toxic side effect of adjuvant chemotherapy, while only a fraction of them will ultimately benefit from it (EBCTCG, 2011). According to the EBTCG meta-analyses, ER status has no impact on proportional reduction with chemotherapy in breast cancer recurrence and death (EBCTCG, 2005; EBCTCG, 2012). On the contrary, the TAILORx trial showed that patients with ER/PR-positive, HER2-negative and node-negative cancer in low risk of recurrence can safely avoid chemotherapy (Sparano et al., 2015). Meanwhile, data from several studies indicated that ER-positive patients have worse response to chemotherapy both in the adjuvant and neoadjuvant settings, compared with ER-negative patients (Montagna et al., 2010).

The strong prognostic performance of the 12-gene signature facilitates potential clinical application in several clinical important settings. As aforementioned, the heterogeneity existing in breast tumors might result in the overtreatment of chemotherapy for early-stage patients, to some extent. The 12-gene signature offers an opportunity for selecting the optimal therapy for early-stage patients, avoiding unnecessary chemotherapy. For advanced and metastatic tumors, the 12-gene signature associated with immune response might be used to identify patients who might benefit from intensify immunotherapies that have shown so much promise in recalcitrant diseases. When compared with a clinical applicable and commercialized model, our signature achieved higher accuracy in predicting OS than Oncotype Dx for ER/PR-positive, HER2-negative, node-negative, or one to three involved lymph nodes breast cancer patients from the full TCGA cohort. Meanwhile, compared with other immune-related signatures, our signature showed a better performance than Teschendorff's 6-gene model and Bianchini's 15-gene model in predicting OS of breast cancer patients from the full TCGA cohort. We further leveraged the complementary value of the immune signature and clinical factors, and found that combining both could provide a more accurate estimation of OS in breast cancer.

The prognostic signature related to the tumor immune microenvironment may hold great promise for identifying novel biomarkers and improving patient management in the era of immunotherapy. As described above, the growth and invasiveness of breast cancer are affected by the presence of various cells in TME. Several recent studies reported that the extent of immune infiltration in TME is associated with breast cancer prognosis. We found higher levels of M2 macrophage infiltration in the high immune risk groups with relatively worse prognosis. M2 macrophages, acting as anti-inflammatory and pro-tumor, are increasingly recognized as contributors to metastatic progression of breast cancer and correlated with poor prognosis (Chowdhury et al., 2019; Little et al., 2019). These are all hints of potential therapeutic opportunities by regulation of M_2_ macrophage-induced migratory and invasive response in breast cancer. In addition, we also note that most genes involved in our signature are relevant to immune response, which may affect the response to immunotherapies. The direction of this effect is difficult to predict, as either increased or decreased immune response may facilitate either more or less immunotherapy response. Taken together, dysregulated immune contexture might account for the survival differences observed between patient groups defined by our signature. Clinical integration of 12-gene signature needs to be tested directly, though appears promising from these initial results.

There are still some limitations in this initial work. First, the patient cohorts were retrospective, therefore these findings must be validated prospectively for further confirmation. Second, we evaluated the performance of the signature in each molecular subtype from the full TCGA cohort lacking further validation, owing to limited patient number in validation datasets. Third, microarray and RNA-seq results are susceptible to bioinformatic parameters that may vary among clinical programs, although we tried to include internal and external datasets for more rigorous validation of our signature.

## Conclusion

In summary, we have performed an immune-related prognostic analysis in breast cancer, resulting in an independently validated 12-gene signature, as well as identification of multiple genes associated with immune infiltration for further study. Stratified analysis further revealed that the 12-gene signature performed well in clinical defined subgroups. Furthermore, the complementary value of the immune-related signature with clinical factors achieved a better accurate stratification of OS in breast cancer patients. Thus, the 12-gene signature is a promising prognostic biomarker and could be a useful predictive tool to select patients who might benefit from immunotherapy.

## Data Availability Statement

The data used and analyzed during the current study are available from UCSC Xena (https://xena.ucsc.edu/) and Gene Expression Omnibus (GEO) (https://www.ncbi.nlm.nih.gov/geo/).

## Author Contributions

Conception and design: PX, JH and HZ. Data collection: YM, SY and RA. Data analysis and interpretation: PX, JH and HZ. Manuscript writing: PX and HZ. Final approval of manuscript: All authors.

## Conflict of Interest

The authors declare that the research was conducted in the absence of any commercial or financial relationships that could be construed as a potential conflict of interest.
